# Suspected Neostigmine-Associated Bronchospasm Complicated by Pulmonary Edema During General Anesthesia: A Case Report

**DOI:** 10.7759/cureus.108996

**Published:** 2026-05-16

**Authors:** Moe Sato, Keiko Haraguchi-Suzuki, Saki Saito, Takashi Suto, Shigeru Saito

**Affiliations:** 1 Department of Anesthesiology, Gunma University Hospital, Maebashi, JPN; 2 Department of Intensive Care Unit, Gunma University Hospital, Maebashi, JPN

**Keywords:** bronchospasm, general anesthesia, hypoxia, negative pressure pulmonary edema, neostigmine

## Abstract

Neostigmine inhibits cholinesterase activity and increases acetylcholine at the neuromuscular junction. Neostigmine is thus administered as an antagonist for non-depolarizing muscle relaxants during general anesthesia. However, neostigmine-induced pulmonary edema is extremely rare, making its diagnosis and appropriate treatment in the perioperative period challenging for anesthesiologists. A middle-aged obese woman underwent surgery for ptosis under general anesthesia because she was allergic to local anesthetics. At the end of surgery, she developed severe hypoxia (peripheral oxygen saturation (SpO_2_) 47%) after administration of neostigmine, and before tracheal extubation. Salbutamol sulfate, a beta2 adrenergic receptor stimulant, was administered through her tracheal tube, which was suspected of bronchospasm, and anesthesia was reintroduced. Chest X-ray showed severe lung infiltration bilaterally, suggestive of negative pressure pulmonary edema. Following improvement of oxygenation by positive pressure ventilation with a high positive end-expiratory pressure (PEEP), her trachea was extubated under deep anesthesia, and manual ventilation was performed until recovery of spontaneous respiration. This report details the management of pulmonary edema that developed acutely after administration of neostigmine during general anesthesia and discusses the cause of hypoxia in our case.

## Introduction

The occurrence of hypoxia during general anesthesia is relatively common; however, the presence of this symptom indicates various potentially fatal conditions. Hypoxia that develops suddenly after reversal of neuromuscular blockade during general anesthesia may result from causes such as anaphylaxis, aspiration, and cardiogenic or non-cardiogenic pulmonary edema. Negative pressure pulmonary edema (NPPE), a type of non-cardiogenic pulmonary edema, is a possible cause of hypoxia, with an estimated incidence of only 0.1% during general anesthesia [1]. A strong inspiratory effort in the presence of airway obstruction, such as due to laryngospasm, decreases intrathoracic pressure and increases venous return, which might result in extravasation of fluid from pulmonary capillaries to the interstitium and alveoli, leading to impaired gas exchange. Simultaneous activation of the sympathetic nerve system seems to worsen the pathophysiology by causing constriction of peripheral vessels, thus increasing venous return [2,3]. Hypoxia caused by NPPE typically improves within 24-48 hours with supportive care, without administration of diuretics, suggesting that NPPE-induced hypoxia is a reversible condition [2,4]. On the other hand, mortality due to NPPE has been reported, including in healthy young patients [5].

Neostigmine increases acetylcholine concentration at the neuromuscular junction through inhibition of cholinesterase activity, thus competitively antagonizing non-depolarizing neuromuscular relaxants [6]. Since acetylcholine also has muscarinic effects, one of which is bronchoconstriction, neostigmine should be co-administered with a muscarinic acetylcholine receptor antagonist such as atropine and should not be administered to patients with a medical history of asthma [7]. When bronchospasm occurs during general anesthesia, it causes an increase in airway pressure and a decrease in tidal volume, which is visualized as a prolonged expiratory upstroke on capnometry [8]. This is typically treated with a beta2 adrenergic receptor agonist and muscarinic acetylcholine receptor antagonist, along with a higher inhaled oxygen concentration and administration of inhalational anesthetics [9]. Importantly, severe bronchospasm has the potential to cause NPPE through the generation of negative intrathoracic pressure, although NPPE caused by bronchospasm is only rarely recognized, since the resultant hypoxia can be masked by oxygen therapy [10-12]. We present here a case of suspected neostigmine-associated bronchospasm complicated by pulmonary edema and its subsequent management.

## Case presentation

A 44-year-old woman, 161 cm tall, weighing 90 kg (body mass index 34.72 kg/m2), was scheduled for ptosis surgery under general anesthesia since she had a medical history of anaphylaxis to local anesthetics. She had undergone a cesarean section under regional anesthesia using lidocaine at the age of 21 years, at which time she developed cardiopulmonary arrest that was suspected to have been caused by anaphylaxis. At the age of 29 years, she underwent cervical conization under regional anesthesia using lidocaine at another hospital, and she reportedly lost consciousness during the procedure. On the other hand, she had undergone removal of an atheroma under general anesthesia nine months prior to her current surgery with an uneventful perioperative course. Hence, the ptosis surgery was scheduled to be performed under general anesthesia.

**Figure 1:**
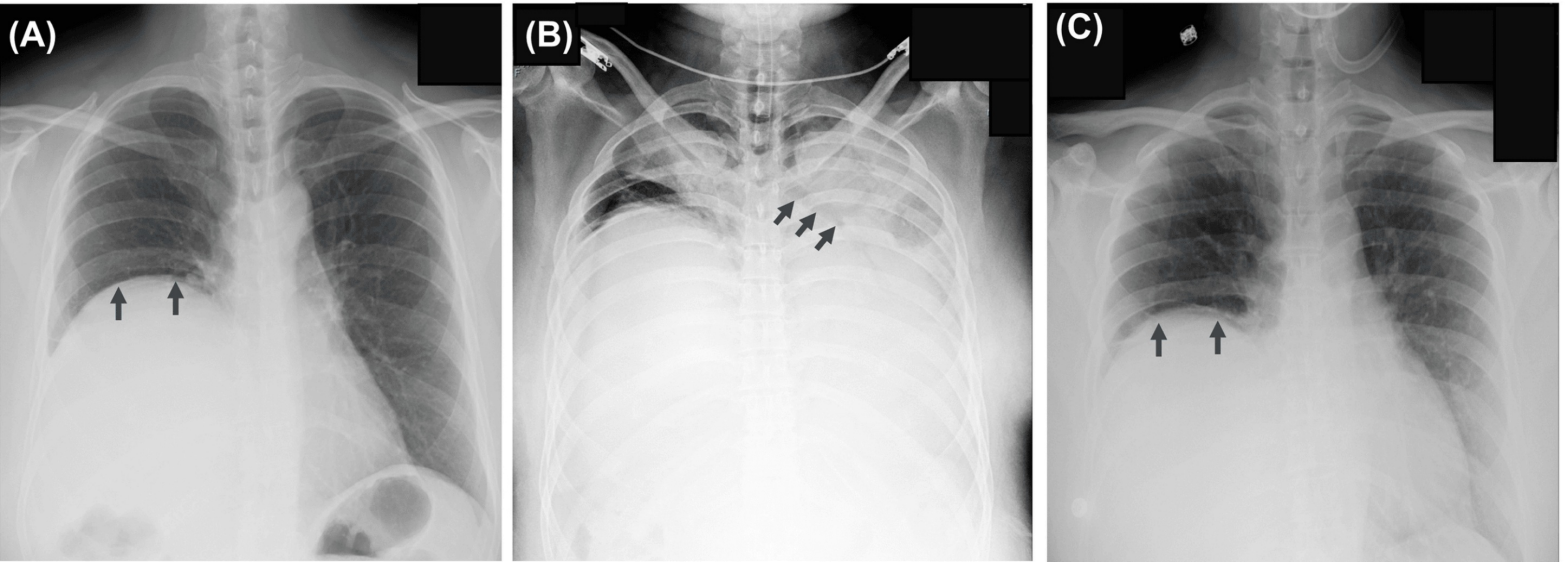
Chest X-ray during perioperative period (A) Preoperative chest X-ray showed elevation of the right hemidiaphragm, suggesting possible phrenic nerve paralysis. No obvious abnormality was observed in the lungs. (B) A chest X-ray obtained immediately postoperatively showed bilateral infiltrates with air bronchograms, suggesting possible pulmonary edema. (C) A chest X-ray obtained on postoperative day 1 showed that the infiltrates in the lung fields had largely resolved, although they persisted in the left lower lung field. Arrows in (A) and (C) indicate elevation of the right hemidiaphragm. Arrows in (B) indicate an air bronchogram.

Her medical history was significant for anxiety and diabetes. She continued taking medications perioperatively, including the benzodiazepine anxiolytics tofisopam and clonazepam, as well as the selective serotonin reuptake inhibitor paroxetine. In contrast, the biguanide antidiabetic drug metformin was withheld perioperatively. She had many allergies, including to fish, milk, alcohol, and crabs, in addition to lidocaine. Preoperative blood tests showed anemia (Hb 10 g/dL, reference range 13.7-16.8 g/dL). On chest X-ray, her right hemidiaphragm was elevated without prolapse of abdominal organs, indicating the possibility of phrenic nerve paralysis (Figure 1A).

When contacted, the previous hospital where the patient had undergone atheroma removal also reported the same observation. Her electrocardiogram showed a heart rate of 92 beats/min and a normal sinus rhythm. She did not have any evidence of heart disease and had no difficulty in her activities of daily living. On entering the operating room, her vital signs indicated a heart rate of 72 beats/min, blood pressure of 129/72 mmHg, and peripheral oxygen saturation (SpO2) of 97%. Since the patient was obese, preoxygenation with 100% oxygen for 5 minutes and rapid sequence induction were performed following administration of 80 mg propofol, 65 mg rocuronium, 0.05 mg fentanyl, 0.15 μg/kg/min remifentanil, and 5% sevoflurane. Since neuromuscular blockade was required only for intubation and not for the ptosis surgery itself, quantitative neuromuscular monitoring was not used in this case. Anesthesia was maintained with oxygen, air, 1.5% sevoflurane, and a 0.15 μg/kg/min remifentanil infusion.

The surgery was performed uneventfully with a surgical time of 79 minutes, minimal blood loss, and an infusion volume of 650 ml. Following recovery of spontaneous respiration, 2.5 mg of neostigmine combined with 1 mg of atropine was administered to antagonize the muscle relaxant. Approximately 2 minutes after administration of neostigmine, the patient started bucking slightly, and her SpO2 suddenly decreased from 100% to 47% within approximately 30 seconds, with an increase in airway pressure from 16 to 40 cmH2O under administration of 100% oxygen. A minimal tidal volume was obtained with both manual and artificial ventilation (pressure-controlled assist/control ventilation), and with prolonged expiratory upstroke on capnography. Airway and ventilator circuit patency were checked, and no obstruction or kinking was found. In addition, no secretions or aspirated material were observed during suctioning of the endotracheal tube. At this time, her blood pressure increased slightly from 105/59 to 120/85 mmHg. Her electrocardiogram revealed sinus rhythm with no ST changes, and her heart rate increased from 55 to 85 beats/min. No cutaneous signs suggestive of anaphylaxis were observed. Although wheezing was not audible on chest auscultation, salbutamol sulfate, a beta2 adrenergic receptor stimulant, was administered through the tracheal tube based on the suspicion of bronchospasm.

Subsequently, her SpO2 increased to 60% with a decrease in airway pressure to 26 cmH2O and an increase in tidal volume to about 300 ml. Anesthesia was reintroduced by administration of 120 mg propofol and 3% sevoflurane. An arterial line was then inserted into the radial artery to monitor oxygenation. Although we did not detect coarse crackles on auscultation or observe frothy sputum during positive pressure ventilation, a chest X-ray taken in the operating room showed bilateral pulmonary infiltrates with air bronchograms, which were observed, suggesting possible bronchospasm-induced pulmonary edema (Figure 1B). At this stage, artificial ventilation (pressure-controlled assist/control ventilation) was performed with positive end-expiratory pressure (PEEP) of 10 cmH2O under 3% sevoflurane anesthesia.

**Figure 2:**
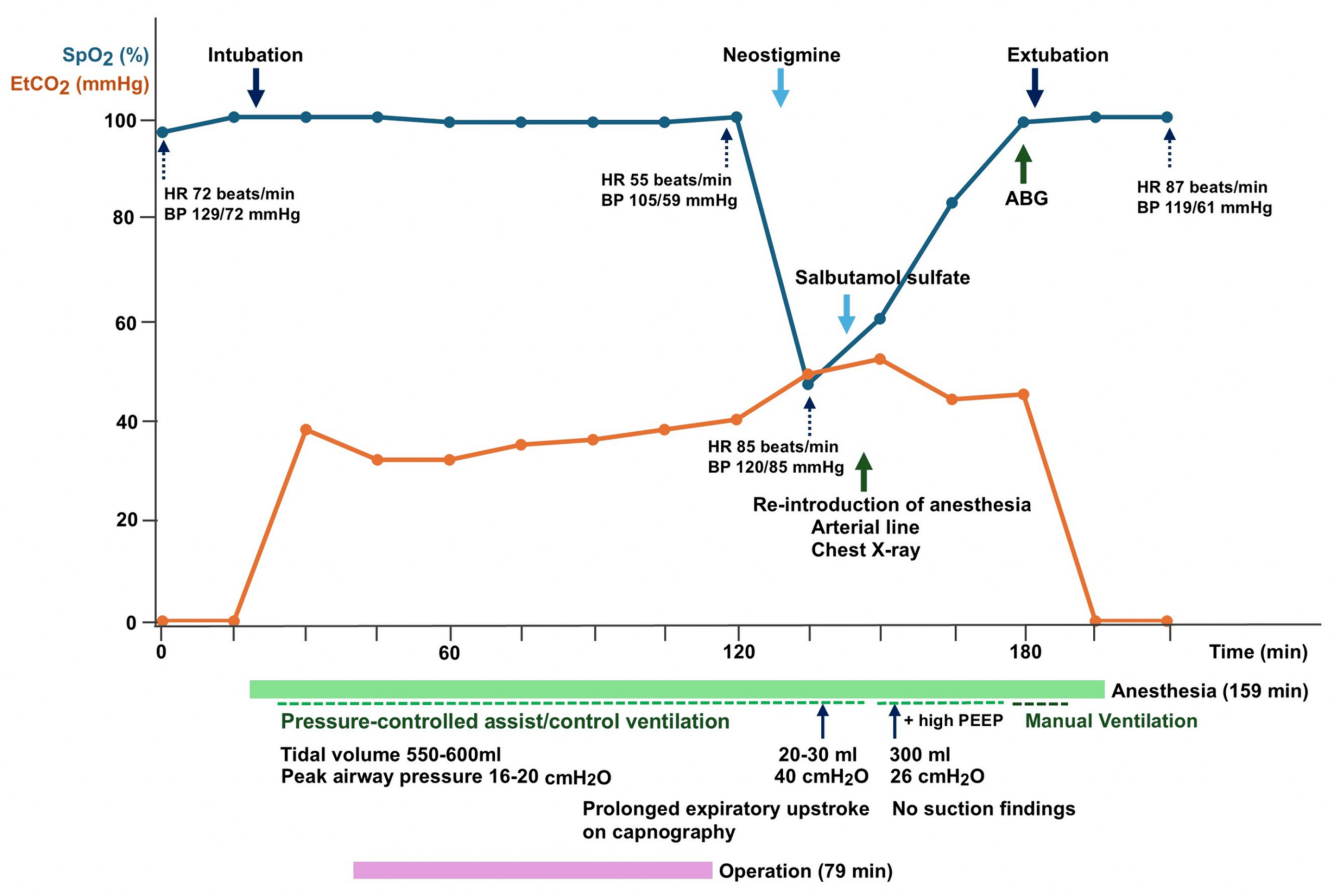
Timeline of general anesthesia A timeline graph of general anesthesia for ptosis surgery illustrating key anesthetic events (intubation, neostigmine administration, salbutamol sulfate administration, and extubation) alongside vital signs (heart rate, blood pressure, SpO₂, and EtCO₂). Respiratory parameters, including ventilation mode, tidal volume, peak airway pressure, and capnography findings, are annotated below the timeline at corresponding time points. HR: heart rate; BP: blood pressure; ABG: arterial blood gas; PEEP: positive end-expiratory pressure; SpO_2_: peripheral oxygen saturation; EtCO_2_: end-tidal carbon dioxide

Thirty minutes after restarting artificial ventilation (pressure-controlled assist/control ventilation) with a high PEEP, her SpO2 increased to 99%. Arterial blood gas analysis indicated a pH of 7.447, partial pressure of oxygen (pO2) of 405 mmHg, and partial pressure of carbon dioxide (pCO2) of 30.8 mmHg under administration of 100% oxygen, indicating improvement of oxygenation. To avoid airway issues, her trachea was extubated under 3% sevoflurane anesthesia while she was on pressure-controlled assist/control ventilation, followed by manual ventilation with 100% oxygen for 9 minutes until recovery of spontaneous respiration. We also administered 6.6 mg dexamethasone to inhibit the recurrence of bronchospasm. The patient recovered spontaneous respiration after extubation, with no audible rales or rhonchi on auscultation. Since respiratory distress and hypoxia were not observed under spontaneous respiration, she was transferred from the operating room to the intensive care unit (ICU). At this time, she was conscious and could follow instructions, such as shaking and releasing her hands. Her vital signs indicated a heart rate of 87 beats/min, blood pressure of 119/61 mmHg, and SpO2 of 99%. The duration of anesthesia was 159 minutes (Figure 2).

She was observed in the ICU with monitoring of her vital signs and was administered oxygen until the following morning. Echocardiography performed after admission to the ICU revealed no evidence of cardiac failure, suggesting that the patient developed non-cardiogenic pulmonary edema rather than cardiogenic pulmonary edema. Chest X-ray on postoperative day 1 showed improvement of infiltrative shadows in the lung (Figure 1C). Her respiratory status remained stable, and she was discharged from the ICU on postoperative day 1. Her subsequent postoperative course was uneventful, and she was discharged from the hospital on postoperative day 3.

## Discussion

We experienced a case of suspected neostigmine-associated bronchospasm complicated by pulmonary edema under general anesthesia in a middle-aged, obese woman. Since the patient did not have heart disease and was not given excessive intravenous infusions during anesthesia, she was considered to have developed non-cardiogenic pulmonary edema secondary to bronchospasm following neostigmine administration (Table 1).

**Table 1:** Differential diagnoses in the present case, differentiating non-cardiogenic pulmonary edema from cardiogenic pulmonary edema.

Diagnosis	Findings in the present case
Cardiogenic pulmonary edema	
Heart failure	Normal cardiac function
Valvular heart disease	No valvular disease
Volume overload during anesthesia	No volume overload during anesthesia
Non-cardiogenic pulmonary edema	
Acute Respiratory Distress Syndrome (ARDS)	No inflammatory symptoms
Sepsis	No infectious symptoms
Transfusion-Related Acute Lung Injury (TRALI)	No blood transfusion
Anaphylaxis	Bronchospasm, but no rash or hypotension
Negative Pressure Pulmonary Edema (NPPE)	Bronchospasm in a relatively young obese patient, no tube obstruction or crackles, but improvement with positive pressure ventilation

The patient had a medical history of many allergies. Since neostigmine is reportedly less likely to cause anaphylaxis as compared to sugammadex, we administered neostigmine to antagonize neuromuscular blockade in this patient [13-15]. Although we did suspect anaphylaxis in this case, she did not have any specific symptoms of anaphylaxis, such as cutaneous signs or hypotension, other than bronchospasm. Additionally, since her hypoxia improved immediately with positive pressure ventilation and a high PEEP without administration of epinephrine or antihistaminic drugs, the pulmonary edema observed in this case was not likely to have been caused by anaphylaxis-related bronchospasm (Table 1). However, in the future, this patient should receive skin tests for allergy to neostigmine before any further application of general anesthesia.

Few previous reports have indicated a correlation between the development of non-cardiogenic pulmonary edema and the administration of neostigmine. Zhang et al. reported a 12-year-old boy who developed non-cardiogenic pulmonary edema after administration of neostigmine following island skin flap surgery on the right upper limb [16]. Neostigmine is known to induce upper airway obstruction through impairing the function of upper airway dilator muscles, such as the genioglossus muscle [17,18]. Thus, although rare, administration of neostigmine can potentially cause an NPPE-like pathological condition. However, the above-reported case of pulmonary edema developed after extubation, while our patient developed pulmonary edema before extubation. Furthermore, her tracheal tube was neither bitten nor obstructed by secretions, indicating that tube obstruction was unlikely and that the patency of the upper airway was maintained.

In our case, bronchospasm was successfully treated with the administration of a beta2 adrenergic receptor agonist, although wheezing was not audible on auscultation, probably because of obesity and the thickness of her chest wall. Although our patient did not have a medical history of asthma, increased acetylcholine concentrations due to neostigmine administration might have been the cause of the bronchospasm, considering the timing of its onset. We used the Naranjo probability scale, which indicated a probable adverse drug reaction [19]. On the other hand, we could not completely rule out airway stimulation by the endotracheal tube as a cause of the bronchospasm, because the patient exhibited slight bucking just before the development of hypoxia. In a previous report, NPPE was caused by bronchospasm with wheezing after extubation following gastrectomy in an elderly man [11]. In cases of severe bronchospasm in healthy young adults prior to extubation, NPPE may be caused by strong inspiratory effort generating markedly negative intrathoracic pressure [2].

A 25-year-old woman who underwent thyroidectomy reportedly developed probable laryngospasm-induced NPPE, accompanied by pink frothy sputum and coarse crackles immediately after extubation. She required re-intubation and assisted ventilation with continuous positive airway pressure (CPAP) for 2 hours, along with administration of diuretics [20]. In contrast to that reported case, our patient exhibited neither sputum production nor crackles, and the hypoxia improved more rapidly with positive-pressure ventilation using high PEEP, without the use of diuretics. Therefore, NPPE may have been caused by either severe bronchospasm before extubation or laryngospasm after extubation, and its severity may depend on the degree of airway obstruction. Furthermore, although a direct relationship between diaphragmatic palsy and the development of NPPE is not known to our knowledge, compensatory use of other respiratory muscles may generate a strong inspiratory effort in cases of hemi-diaphragmatic palsy.

Additionally, although we confirmed recovery of spontaneous respiration before administration of neostigmine in our patient, use of neuromuscular monitoring in this patient, if performed, might have indicated that neostigmine administration was not required. Neuromuscular monitoring is also important in such a high-risk patient.

## Conclusions

Due to its rarity, the management of hypoxia caused by probable neostigmine-associated bronchospasm complicated by acute non-cardiogenic pulmonary edema during general anesthesia can be challenging for anesthesiologists. Although the use of neostigmine as a reversal agent for neuromuscular blockade appears to have decreased in recent clinical practice, it remains useful in patients with a history of sugammadex-induced anaphylaxis. We learned from this case the importance of quantitative neuromuscular monitoring, the cautious administration of neostigmine in high-risk patients, and the prompt recognition and treatment of bronchospasm and NPPE.
